# Supplementary material for: Bio-fabrication of stem-cell-incorporated corneal epithelial and stromal equivalents from silk fibroin and gelatin-based biomaterial for canine corneal regeneration
Source: PLoS One. 2022 Feb 4;17(2):e0263141. doi: 10.1371/journal.pone.0263141 (PMC8815981; doi:10.1371/journal.pone.0263141)
Supplement: S1 Table — Breed, gender and age of the subjects. (DOCX) [file pone.0263141.s001.docx]

**S1 Table. General information of the subjects.** Breed, gender and age of the subjects.

| **Sample number** | **Breed** | **Gender** | **Age** |
| --- | --- | --- | --- |
| 1. | Golden retriever | Male | 7 years |
| 2. | Poodle | Female | 7 years |
| 3. | Mixed breed | Female | 5 years |
| 4. | Mixed breed | Male | 1 year |
| 5. | Chihuahua | Female | 1 year |
| 6. | Mixed breed | Male | 7 years |
| 7. | Pit Bull | Female | 7 years |
| 8. | Shi Tzu | Male | 5 years |
| 9. | Mixed breed | Female | 6 years |
| 10. | Mixed breed | Female | 7 years |
| 11. | Pit Bull | Male | 7 years |
| 12. | Shih tzu | Female | 5 years |
| 13. | Shih tzu | Male | 7 years |
| 14. | Shih tzu | Female | 4 years |
| 15. | Siberian Husky | Female | 7 years |
