# Supplementary material for: Bio-fabrication of stem-cell-incorporated corneal epithelial and stromal equivalents from silk fibroin and gelatin-based biomaterial for canine corneal regeneration
Source: PLoS One. 2022 Feb 4;17(2):e0263141. doi: 10.1371/journal.pone.0263141 (PMC8815981; doi:10.1371/journal.pone.0263141)
Supplement: Attachment — Submitted filename: Response to Reviewers.docx [file pone.0263141.s002.docx]

PONE-D-21-30098

Bio-fabrication of stem cell-incorporated corneal equivalents from silk fibroin and gelatin-based biomaterial for canine corneal regeneration

PLOS ONE

Dear Dr. Sawangmake,

Thank you for submitting your manuscript to PLOS ONE. After careful consideration, we feel that it has merit but does not fully meet PLOS ONE’s publication criteria as it currently stands. Therefore, we invite you to submit a revised version of the manuscript that addresses the points raised during the review process.

Please submit your revised manuscript by Jan 08 2022 11:59PM. If you will need more time than this to complete your revisions, please reply to this message or contact the journal office at [plosone@plos.org](mailto:plosone@plos.org). When you're ready to submit your revision, log on to <https://www.editorialmanager.com/pone/> and select the 'Submissions Needing Revision' folder to locate your manuscript file.

Please include the following items when submitting your revised manuscript:

- A rebuttal letter that responds to each point raised by the academic editor and reviewer(s). You should upload this letter as a separate file labeled 'Response to Reviewers'.

*Response: Rebuttal letter including point-by-point responses to the reviewer and editor comments was completed and uploaded.*

- A marked-up copy of your manuscript that highlights changes made to the original version. You should upload this as a separate file labeled 'Revised Manuscript with Track Changes'.

*Response: All changes to the manuscript were highlighted in blue color, and the manuscript file with the highlight was uploaded.*

- An unmarked version of your revised paper without tracked changes. You should upload this as a separate file labeled 'Manuscript'.

*Response: An unmarked version of the revised paper was uploaded.*

If you would like to make changes to your financial disclosure, please include your updated statement in your cover letter. Guidelines for resubmitting your figure files are available below the reviewer comments at the end of this letter.

If applicable, we recommend that you deposit your laboratory protocols in [protocols.io](http://protocols.io) to enhance the reproducibility of your results. Protocols.io assigns your protocol its own identifier (DOI) so that it can be cited independently in the future. For instructions see: <https://journals.plos.org/plosone/s/submission-guidelines#loc-laboratory-protocols>. Additionally, PLOS ONE offers an option for publishing peer-reviewed Lab Protocol articles, which describe protocols hosted on [protocols.io](http://protocols.io). Read more information on sharing protocols at <https://plos.org/protocols?utm_medium=editorial-email&utm_source=authorletters&utm_campaign=protocols>.

We look forward to receiving your revised manuscript.

Kind regards,

Panayiotis Maghsoudlou
Academic Editor
PLOS ONE

Journal Requirements:

When submitting your revision, we need you to address these additional requirements.

1. Please ensure that your manuscript meets PLOS ONE's style requirements, including those for file naming. The PLOS ONE style templates can be found at
   <https://journals.plos.org/plosone/s/file?id=wjVg/PLOSOne_formatting_sample_main_body.pdf> and 
   <https://journals.plos.org/plosone/s/file?id=ba62/PLOSOne_formatting_sample_title_authors_affiliations.pdf>

*Response: We have checked the guideline. The manuscript complies with the format requirement.*

2. We suggest you thoroughly copyedit your manuscript for language usage, spelling, and grammar. If you do not know anyone who can help you do this, you may wish to consider employing a professional scientific editing service.

Whilst you may use any professional scientific editing service of your choice, PLOS has partnered with both American Journal Experts (AJE) and Editage to provide discounted services to PLOS authors. Both organizations have experience helping authors meet PLOS guidelines and can provide language editing, translation, manuscript formatting, and figure formatting to ensure your manuscript meets our submission guidelines. To take advantage of our partnership with AJE, visit the AJE website ([http://aje.com/go/plos](http://aje.com/go/plos)) for a 15% discount off AJE services. To take advantage of our partnership with Editage, visit the Editage website ([www.editage.com](http://www.editage.com)) and enter referral code PLOSEDIT for a 15% discount off Editage services.  If the PLOS editorial team finds any language issues in text that either AJE or Editage has edited, the service provider will re-edit the text for free.

Upon resubmission, please provide the following:
 
The name of the colleague or the details of the professional service that edited your manuscript

*Response: The manuscript was proofread by a professional copyediting service, ProofreadingService.com.*

A copy of your manuscript showing your changes by either highlighting them or using track changes (uploaded as a *supporting information* file)

*Response: All changes to the manuscript were highlighted in blue color, and the manuscript file with the highlight was uploaded as a supporting information file.*

A clean copy of the edited manuscript (uploaded as the new *manuscript* file)

*Response: An unmarked version of the revised paper was uploaded as the new manuscript file.*

[Note: HTML markup is below. Please do not edit.]

Reviewers' comments:

Reviewer's Responses to Questions

**Comments to the Author**

1. Is the manuscript technically sound, and do the data support the conclusions?

The manuscript must describe a technically sound piece of scientific research with data that supports the conclusions. Experiments must have been conducted rigorously, with appropriate controls, replication, and sample sizes. The conclusions must be drawn appropriately based on the data presented.

Reviewer #1: Yes

Reviewer #2: Yes

Reviewer #3: Yes

2. Has the statistical analysis been performed appropriately and rigorously?

Reviewer #1: Yes

Reviewer #2: Yes

Reviewer #3: Yes

3. Have the authors made all data underlying the findings in their manuscript fully available?

The [PLOS Data policy](http://www.plosone.org/static/policies.action#sharing) requires authors to make all data underlying the findings described in their manuscript fully available without restriction, with rare exception (please refer to the Data Availability Statement in the manuscript PDF file). The data should be provided as part of the manuscript or its supporting information, or deposited to a public repository. For example, in addition to summary statistics, the data points behind means, medians and variance measures should be available. If there are restrictions on publicly sharing data—e.g. participant privacy or use of data from a third party—those must be specified.

Reviewer #1: Yes

Reviewer #2: Yes

Reviewer #3: Yes

4. Is the manuscript presented in an intelligible fashion and written in standard English?

PLOS ONE does not copyedit accepted manuscripts, so the language in submitted articles must be clear, correct, and unambiguous. Any typographical or grammatical errors should be corrected at revision, so please note any specific errors here.

Reviewer #1: No

Reviewer #2: No

Reviewer #3: Yes

5. Review Comments to the Author

Please use the space provided to explain your answers to the questions above. You may also include additional comments for the author, including concerns about dual publication, research ethics, or publication ethics. (Please upload your review as an attachment if it exceeds 20,000 characters)

Reviewer #1: he authors should check their manuscript for consistency, for instance ml and mL are both used. Also, numbers are written in full and as numbers; please write numbers until twenty in full and higer in numbers.

*Response: We have edited ml to mL, and all numerals have been written as numbers for consistency.*

Language remarks:
45: I have been told never to use abbreviations in the abstract of an article. Even more, they are not defined upon their first use in the abstract.

*Response: We have defined all of the abbreviations in the abstract.*

76: corneal grafts

*Response: We have corrected it as suggested (line 75).*

83-84: the use of the ex. Abbreviation is not common. Please use alternatives such as: for example, e.g. etc. (also on line 91)

*Response: We have changed to e.g. (line 83).*

92: transparency

*Response: We have corrected it as suggested (line 92).*

107: promising: remove

*Response: We have removed the aforementioned word as suggested.*

109: “were replenished the knowledge” ◊ change

*Response: The sentence was changed from “* were replenished the knowledge*” to “contribute to the body of knowledge on cell culture in veterinary medicine and are worth further experimentation*” *(line 112-113).*

143: remove a before 2 ml

*Response: We have removed a before 2 mL (line 140).*

302: In brief: … ◊ change the structure of the sentence.

*Response: We have changed the structure of the sentence (line 301-303).*

345: twice of… use correct English formulation

*Response: We have changed the sentence to “5-min microwave heating (600 w) was performed twice”(line 345).*

361: shield instead of shied

*Response: We have changed this as suggested (line 359).*

415: incorrect use of language

*Response: : We have changed the sentence to “In terms of the SF/G stromal scaffold, the appearance revealed a coin-like, opaque white shape” (line 431-432).*

439 decreased instead of deceased

*Response: We have corrected (line 455).*

530 Pax6 was similar pattern is no correct English.

*Response: We have edited the sentence to “Pax6 expression showed similar pattern to..” (line 549-550).*

645: promote instead of promotes

*Response: We have corrected it as suggested (line 669).*

647: revise sentence

*Response: We have revised the sentence to “Collagen type I (Col-1) which is abundant in corneal stroma..” (line 671).*

720: gelatin containing RGD

*Response: We have changed this as suggested (line 774).*

740: therefor … revise sentence

*Response: The sentence has been revised to “therefore, those makers were validated in this study (line 798-799).*

755: arise from… what is meant here?

*Response: The words have been removed.*

Reviewer #2: Bio-fabrication of stem cell-incorporated corneal equivalents from silk fibroin and gelatin-based biomaterial for canine corneal regeneration

The authors propose a new technology for bio-engineering corneal tissue using silk fibroin/gelatin films and scaffolds as careers to grow, respectively, limbal stem cells and corneal stromal stem cells. LSC seeded films and CSSC seeded scaffolds were assembled using tissue glue. Canine stem cells were used to prepare artificial corneas aimed at treating dogs with corneal blindness.

Abstract. Many abbreviations are used in the abstract (LSC, CSSC..). They should be first shown in full (limbal stem cell…). Otherwise the abstract is difficult to read.

*Response: We have defined all of the abbreviations in the abstract.*

The manuscript should be corrected for language by a native English speaker as many sentences are awkward or difficult to understand.

*Response: The content of the manuscript was proofread by a native English speaker before resubmission, and the corrections were marked in red font color.*

“In humans and animals, corneas consist of 5 recognized layers including 3 cellular layers (epithelium, stroma, and endothelium) and 2 acellular layers (Bowman’s layer and Descemet’s membrane).”
Descemet’s membrane is not a corneal layer. It is the corneal endothelium basement membrane.

*Response: We acknowledge your concern and would add the information in discussion part.*

*“Canine cornea consists of 3 cellular layers (corneal epithelium, corneal stroma, and corneal endothelium) and one interface called Descemet’s membrane, which is a dense, thick, basement membrane for the corneal endothelium (6, 29–31)” (line 692-695).*

“LESCs and CSSCs have been progressed in stem cell properties ex. clonal growth in vitro, extended lifespan, and the ability of differentiation in particular keratocytes.”
What does this sentence mean?

*Response: The sentence has been revised to*

*“LESCs and CSSCs have stem cell properties, e.g. clonal growth in vitro, extended lifespan, and the ability of differentiation into corneal cells (13)”( line 83-85).*

“Various materials have been used to fabricate three-dimensional (3-D) biocompatible scaffolds such as silk protein.”
The main material used as a scaffold is collagen (see Fagerholm, P., Lagali, N.S., Merrett, K. et al., 2010. A biosynthetic alternative to human donor tissue for inducing corneal regeneration: 24-month follow-up of a Phase 1 clinical study. Sci. Transl. Med. Tidu, A., Ghoubay-Benallaoua, D., Teulon, C., et al., 2018. Highly concentrated collagen solutions leading to transparent scaffolds of controlled three-dimensional organizations for corneal epithelial cell colonization. Biomater. Sci…).

*Response: We accept the advantage of collagen which is the most abundant of ECM in cornea and biomimetic corneal stroma. However, canine ocular scaffold points to have high mechanical toughness because of the activity and behavior of the animals. Silk fibroin (SF) presents superior mechanical toughness and slow degradation. As a result of those reasons, SF was preferred in the study (Chen et al., 2018* *doi:10.1088/1748-605X/aa92d2). We would add the information in the discussion part.*

*“Natural materials have been used to fabricate three-dimensional (3-D) biocompatible scaffolds including collagen, silk fibroin and gelatin. Collagen have been widely accepted as a corneal scaffold (39, 40) as well as silk fibroin (18, 19, 41, 42). However, the property of SF shows superior mechanical toughness and slow degradation which are appropriate for the corneal scaffold of the high activity animals (14). As a result, SF/G was selected…” (line737-742).*

Preparation of gelatin. The authors should provide the characteristics of the gelatin used in their study (specie, preparation process, chemical characteristics).

*Response: We have descripted the characteristics of gelatin as suggested. Gelatin was obtained from Nitta Gelatin Inc., Japan. Type A gelatin produced from porcine skin collagen via acidic treatment (isoelectric point, pl~7-9) (line 133- 134).*

“Unilateral/Bilateral corneas were obtained from fifteen cadaveric healthy dog eyes”
Please provide the spicy.

*Response: We have added the information in supporting information table (S1 table).*

Figure legends are lacking.

Fig. 1. Images of immunofluorescence of the various markers used to characterize stem cells would be needed. Although informative, RT-PCR graphs do not allow protein staining intensity and localization to be observed.

*Response: To address this issue, in our study we focus on how to generate canine corneal epithelial and stromal equivalents. Accordingly, cell characterization is one part of our work that includes specific gene and protein expression that have been mostly reported to characterize LESCs and CSSC in many studies (Rohaina et al., 2014 doi.org/10.1016/j.trsl.2013.11.004, Guo et al., 2020 doi.org/10.1016/j.exer.2020.107915, Funderburgh et al., doi: 10.1096/fj.04-2770fje, Weng et al.,2020 doi.org/10.1186/s40662-020-00217-z). Especially,* P63 and Pax6 *proteins have been used to characterize LESCs and CSSCs in canine respectively (Nam et al., 2015 DOI:10.1111/vop.12241, Kafernic et al.,2020 doi.org/10.1089/scd.2019.0163 and Morita et al., 2015 DOI:10.1111/vop.12147).*

*Moreover, to characterized cCSSCs, we also performed cCSSCs differentiation into keratocytes using keratocyte differentiation media (KDM) in culture plate. The morphology of cCSSCs was changed to keratocyte morphology (stellate formation with numerous cell processes) and fold changes of RT-qPCR presented significate upregulation of keratocyte marker and downregulation of adult stemness-related markers. Data were presented in Fig. 2 and added in the result and discussion part (line 403-418, line 728-735).*

Fig 1B shows a colony of spindle-shaped fibroblasts that are not features of CSSC. Did the authors obtain spheres when growing CSSC? (see Ghoubay-Benallaoua D, et al. Easy xeno-free and feeder-free method for isolating and growing limbal stromal and epithelial stem cells of the human cornea. PLoS One 2017).

*Response: We acknowledge your concern. To address this issue, we studied that research and found some differences of the culture technique for example using different cell species, media, and plate. We compared our cell morphology with the study of Kafernic et al.,2020 doi.org/10.1089/scd.2019.0163 which also used the same species that revealed elongate, fibroblastic morphology. Moreover, CSSCs have been accepted to be MSCs which present indistinguishable fibroblastic morphology (Denu et al., 2016 doi:10.1159/000445096). Sphere-forming culturing is a widely accepted method for the isolation of stem, or progenitor cells (Reynolds et al.,1992, DOI: 10.1126/science.1553558). However, in this study, we isolated cCSSCs from the limbal area where is the stem cell niche and characterized by RT-qPCR in the specific gene and immunocytochemistry of specific protein. Besides, after differentiation into keratocyte, the cell morphology was changed to keratocyte morphology and upregulated of keratocyte markers including Aqp1, Aldh3a1, Kera and Lum. Data were presented in Fig. 2.*

3D colonization of the CSSC seeded scaffolds is an important issue. Fig. 3 does not provide evidence of 3D colonization as SEM is only a surface technique. Please provide cross-sections with the top and bottom surfaces of the scaffold identified.

*Response: To address this issue, in Fig. 4, we aim to show the capacity to adhere the cells in their scaffold, hence we presented the top view in SEM. However, the cross-section of SEM also provided in the Fig. 6 and the cross-section and bottom of cCSSCs seeded scaffold with live/dead staining were added in the Fig. 4E and 4F to illustrate cell adhesion within the scaffold.*

“Cross-sectional figure demonstrated the upper part of cLESCs seeded SF/G corneal film adhered to the lower part of cCSSCs seeded SF/G stromal scaffold, besides the interconnected space was absent.” This sentence is not supported by Fi. 6 which shows a large space between the film and the scaffold (HES).

*Response: To address this issue, the interconnection space of SEM the Fig. 6 was measured by ImageJ and presented 12.71 ± 6.3 µm while the interconnection space of H&E staining in the Fig. 7 showed 108.94 ± 32.8 µm. The different distance appeared 8.5 times. Tissue processing during H&E sectioning process might be the cause of this aspect because it was not operated in SEM protocols. The data were added in discussion part.*

*“The space between cLESCs seeded SF/G corneal films and cCSSCs seeded SF/G corneal scaffold in H&E staining appeared 8.5 times bigger than in SEM. Tissue processing during H&E sectioning process might be the cause of this aspect because it was not operated in SEM protocols” (line 862-865).*

Easy xeno-free and feeder-free method for isolating and growing limbal stromal and epithelial stem cells of the human cornea

FiG. 6 shows the stromal ultrastructure is quite different from the normal corneal stromal ultrastructure. The obtained ultrastructure is not likely to be associated with a high level of transparency as it is completely disorganized.

*Response: We appreciate your concern and totally agree that the ultrastructure was different to normal cornea. To address this issue, in our study, we would like to create the prototype of corneal epithelial and stromal equivalents and provided favorable biocompatibility between cells and scaffold. Besides, the platform of our study about cell isolation, culture, material fabrication would be beneficial in the next study. We have completed the suggestion data how to generate biomimetic corneal stoma using scaffold topography in the discussion part.*

*“Nevertheless, SF/G scaffold created an irregular ultrastructure that was difficult to succeed the biomimetic corneal stroma. To approach multilayered lamellae with orthogonally oriented collagen fibrils, topographical modification of scaffold would be beneficial to regulate the collagen’s direction. For example, a groove topography successfully manifested well-defined lamella collagen orientation and promoted proper ECM including keratan sulfate, lumican, and keratocan (52)” (line 882-888).*

“By our established technique, the stem cell-incorporated corneal equivalents for canine corneal regeneration were successfully generated.”
This sentence is not supported by the data shown.

*Response: We have changed the sentence to “Our established technique successfully generated stem-cell-incorporated corneal epithelial and stromal equivalents for canines” (line 614-615).*

Discussion should mention three major study limitations. First, the normal corneal stromal ultrastructure was not regenerated preventing normal transparency to be reached. Second, the material transparency at various light wavelengths was not assessed. Last no attempt to seed the reconstructed tissue with endothelial cells was made. As a result, the resulting bio-engineered tissue cannot be used for penetrating or endothelial keratoplasty.

*Response: We appreciate your suggestion. We have completed the information in the discussion part.*

*“The transparency of scaffolds could be qualitatively observed by placing the scaffold over colored paper. However, the qualitative degree of transparency, the assessment by spectrophotometer in different wavelengths, was not analyzed in this study (46)” (line 748-751).*

*“Nevertheless, SF/G scaffold created an irregular ultrastructure that was difficult to succeed the biomimetic corneal stroma. To approach multilayered lamellae with orthogonally oriented collagen fibrils, topographical modification of scaffold would be beneficial to regulate the collagen’s direction” (line 882-886).*

*“However, this study produced an insufficient corneal endothelial layer on the posterior side. Corneal endothelium is important to maintain corneal transparency by transporting water out of the stroma, controlling corneal hydration and nutrition (75). Accordingly, corneal epithelial and stromal equivalents could not be manipulated for penetrating or endothelial keratoplasty. To satisfy many clinical applications, 3 cellular layers should be generated in further experiments” (line 889-894).*

Reviewer #3: The manuscript titled, "Bio-fabrication of stem cell-incorporated corneal equivalents from silk fibroin and gelatin-based biomaterial for canine corneal regeneration" describes silk fibroin and gelatin mixed biomaterials as a scaffold material for canine corneal tissue regeneration. Authors prepared silk fibroin and primary limbal epithelial stem cells and corneal stromal stem cells for this study. Additionally, cLESCs and cCSSCs cultured SF/G scaffolds were cultured for up to 28 days and analysed for corneal cell specific markers. The corneal regeneration study is not so many until now and this manuscript is accountable for the cell proliferation and tissue regeneration trial.
However, there are points to be addressed prior to publication.

Major points;
1. Discuss about the advantages of gelatin mixing with the silk fibroin. There looks minimal advantage with the addition of gelatin, as it is soluble to water and the gelatin content in the silk fibroin scaffold would be lower than initial mixing ratio.

*Response: To address this issue, we already completed the information in the discussion part.*

*“SF itself shows a lack of the RDG sequence. Mixing with gelatin-containing RGD can promote cell adhesion capacity; therefore, an RGD-modified process that would result in chemical residues was unnecessary. In addition, incorporation with gelatin was useful to adjust the suitable degradation rate (23). In term of mixing ratio, SF/G (a ratio of 30/70 and final solid concentration of 5% w/w) demonstrated the most cell adhesion capacity compared to 0/100, 50/50, 70/30, and 100/0 in the pilot study” (line 773-779).*

*As a result of gelatin which is a water-soluble protein. In this study, we adopted dehydrothermal technique at 140°C for 72 h under vacuum in a vacuum oven to crosslink SF/G stromal scaffold before using. (Ratanavaraporn et al., 2010, Influences of physical and chemical crosslinking techniques on electrospun type A and B gelatin fiber mats, doi: 10.1016/j.ijbiomac.2010.06.008).*

*In pilot study, SF/G corneal film and stromal scaffold were maintainable their structure in culture media up to 28 days.*

2. In the title, the expression "Corneal equivalents" is inappropriate because this study does not include corneal endothelium. Corneal endothelium is essential for maintaining transparent cornea by pumping out water and dehydrate corneal stroma.

*Response: We acknowledge your concern and have changed from corneal equivalents to corneal epithelial and stomal equivalents. The objective of two-layers generation has been descripted in the discussion part.*

*“Superficial corneal ulcer, deep corneal ulcer, spontaneous chronic corneal ulceration (SCCED), and descemetocele are common types of corneal ulcer that affect the part of the corneal epithelium and stroma (6). Thus, canine corneal epithelial and stromal equivalents were generated as a transplantable graft using cell-incorporated SF/G scaffold” (line 696-700).*

*However, the advantage of corneal epithelium has been provided in discussion part (line 889-894).*

3. Add discussion about the corneal endothelium for corneal equivalent biofabrication in the discussion part.

*Response: We have added in the discussion part (line 889-894).*

*“However, this study produced an insufficient corneal endothelial layer on the posterior side. Corneal endothelium is important to maintain corneal transparency by transporting water out of the stroma, controlling corneal hydration and nutrition (75). Accordingly, corneal epithelial and stromal equivalents could not be manipulated for penetrating or endothelial keratoplasty. To satisfy many clinical applications, 3 cellular layers should be generated in further experiments.”*


4. Figure 7. There looks negligible cell number and collagen amount change during day 14 and day 28. It is recommended to add images of culture day 1 and add quantitative collagen amount data for clear collgen type 1 change with cell culture period.

*Response: To address this issue, this study aim to generate corneal epithelial and stromal equivalents. At day 14 and 28, cCSSCs were anticipated to product collagen type 1 after 4 weeks (Karamichos et al.,* *doi:10.1371/journal.pone.0086260). On the other hand, for corneal transplantation, the regenerative cornea should be transplanted earlier before self-healing process and other complications. Therefore, to assess collagen production at day 14 was performed. The quantitative collogen amount was determined using ImageJ and normalized by the area of scaffold. The result revealed significant difference (p <0.05) between 86.62 ± 2.38% of day 14 and 165.03 ± 9.79% of day 28. The data showed the elevation of collagen production by timeline. However, at day 14, the collagen was produced and has a possibility to use (line 674-678).*

5. line 457 There are only the mechanical properties of the scaffolds. Recommended to add data for canine corneal tissue mechanical properties. Further, add discussion about the mechanical property data of the scaffolds with the native corneal tissues.

*Response: To address this issue, mechanical properties of canine corneal tissue have been studied but determined with secant modulus and tangent modulus (He et al., 2011, doi:https://doi.org/10.1167/iovs.10-5723) and used atomic force microscopy to analyze elastic modulus (Leonard et al., 2019, doi:10.3390/bioengineering7010004), so the data could not be compared. However, the data of the mean secant and tangent modulus have been added in discussion part.*

*“Concerning this, there have been a few studies on mechanical properties of canine cornea. He et al. demonstrated the mean secant modulus and tangent modulus with equivalent loading at 1.05 ± 0.40 and 1.28 ± 0.47 Mpa, respectively. The mean secant modulus increased from 1.07 ± 0.48 MPa at 1% strain to 2.01 ± 0.98 MPa at 5% strain, while the mean tangent modulus increased from 1.28 ± 0.69 to 3.16 ± 0.71 MPa (51)” (line 766-771).*

6. Overall, quantitative result data were not written or included in the results parts. The numbers and statistical significance should be included in the manuscript.

*Response: To address this issue, the quantitative results have been added.*

Minor points;
1. Use subscript for W0, Wt.

*Response: We have followed your advice. (Line 180-181, 194-195)*

2. Use symbol instead of alphabet "X" in the equation.

*Response: We have used the equation tool to display all of the equations.*

3. Add scale bars in the figure legends.

*Response: We have added the scale bars in all of the figure legends.*

4. line 127 molar concentration is missing. : 9.3M LiBr solution

*Response: We have added M as suggested (line 125).*

5. line 154 Detailed description is needed how the air interphase area of scaffolds was removed.

*Response: Because air interface is the area that has a shortage of porosity and obviously presents on the top of the scaffold. To remove air interface, we used microtome blade to slide the upper part of the scaffold after performing dehydrothermal technique. The detail has been added as suggested (line 151-153).*

6. line 155 Please describe in detail how the scaffold was made into 1 mm thickness (include tool, procedure and so on).

*Response: 1 mm thickness of the scaffold was measured by Vernier Caliper and slid the scaffold using microtome blade. Before using, the scaffolds were weighted for estimating the density. The scaffolds in the similar weight were selected for further study. The detail has been added as suggested (line 153-157).*

6. PLOS authors have the option to publish the peer review history of their article ([what does this mean?](https://journals.plos.org/plosone/s/editorial-and-peer-review-process#loc-peer-review-history)). If published, this will include your full peer review and any attached files.


If you choose “no”, your identity will remain anonymous but your review may still be made public.


**Do you want your identity to be public for this peer review?** For information about this choice, including consent withdrawal, please see our [Privacy Policy](https://www.plos.org/privacy-policy).

Reviewer #1: **Yes:** Bert Van den Bogerd

Reviewer #2: No

Reviewer #3: No

[NOTE: If reviewer comments were submitted as an attachment file, they will be attached to this email and accessible via the submission site. Please log into your account, locate the manuscript record, and check for the action link "View Attachments". If this link does not appear, there are no attachment files.]

While revising your submission, please upload your figure files to the Preflight Analysis and Conversion Engine (PACE) digital diagnostic tool, <https://pacev2.apexcovantage.com/>. PACE helps ensure that figures meet PLOS requirements. To use PACE, you must first register as a user. Registration is free. Then, login and navigate to the UPLOAD tab, where you will find detailed instructions on how to use the tool. If you encounter any issues or have any questions when using PACE, please email PLOS at [figures@plos.org](mailto:figures@plos.org). Please note that Supporting Information files do not need this step.
